# Noise-assisted energy transport in electrical oscillator networks with off-diagonal dynamical disorder

**DOI:** 10.1038/srep17339

**Published:** 2015-11-27

**Authors:** Roberto de J. León-Montiel, Mario A. Quiroz-Juárez, Rafael Quintero-Torres, Jorge L. Domínguez-Juárez, Héctor M. Moya-Cessa, Juan P. Torres, José L. Aragón

**Affiliations:** 1 Instituto Nacional de Astrofísica, Óptica y Electrónica, Calle Luis Enrique Erro 1, Santa María Tonantzintla, Puebla CP 72840, México; 2 Department of Chemistry & Biochemistry, University of California San Diego, La Jolla, California 92093, USA; 3 Centro de Física Aplicada y Tecnología Avanzada, Universidad Nacional Autónoma de México, Boulevard Juriquilla 3001, Juriquilla Querétaro 76230, México; 4 Escuela Superior de Ingeniería Mecánica y Eléctrica, Culhuacán. Instituto Politécnico Nacional, Santa Ana 1000, San Francisco Culhuacán 04430, Distrito Federal, México; 5 Cátedras CONACyT, Centro de Física Aplicada y Tecnología Avanzada, Universidad Nacional Autónoma de México, Boulevard Juriquilla 3001, Querétaro 76230, México; 6 ICFO - Institut de Ciències Fotòniques, Mediterranean Technology Park, 08860 Castelldefels (Barcelona), Spain; 7 Department of Signal Theory and Communications, Jordi Girona 1–3, Campus Nord D3, Universitat Politècnica de Catalunya, 08034 Barcelona, Spain

## Abstract

Noise is generally thought as detrimental for energy transport in coupled oscillator networks. However, it has been shown that for certain coherently evolving systems, the presence of noise can enhance, somehow unexpectedly, their transport efficiency; a phenomenon called environment-assisted quantum transport (ENAQT) or dephasing-assisted transport. Here, we report on the experimental observation of such effect in a network of coupled electrical oscillators. We demonstrate that by introducing stochastic fluctuations in one of the couplings of the network, a relative enhancement in the energy transport efficiency of 22.5 ± 3.6% can be observed.

Transport phenomena are ubiquitous throughout different fields of research. Some of the most common examples of transport analysis are seen in the fields of physics, chemistry and biology^1^. In recent years, energy transport assisted by noise^2^,^3^,^4^ has attracted a great deal of attention, partly because of its potential role in the development of future artificial light-harvesting technologies^5^,^6^,^7^. This intriguing phenomenon has theoretically been shown to occur in several quantum^8^,^9^,^10^,^11^,^12^,^13^,^14^ and classical^15^,^16^,^17^,^18^ systems; however, efforts towards its experimental observation had not been presented until very recently. Viciani *et al.*^19^ showed an enhancement in the energy transport of optical fiber cavity networks, where the effect of noise on the system was introduced by averaging the optical response of several network configurations with different cavity-frequency values. In a closely related experiment, Biggerstaff *et al.*^20^ demonstrated an increase in the transport efficiency of a laser-written waveguide network, where decoherence effects were simulated by averaging the output signal of the waveguide array considering different illumination wavelengths. Using the same photonic platform, Caruso *et al.*^21^ observed an enhanced transport efficiency when suppressing interference effects in the transport dynamics of a photonic network. In this experiment, noise was implemented by dynamically modulating the propagation constants of the waveguides, which is the natural way for producing decohering noise, as it has been experimentally demonstrated in the context of quantum random walks^22^,^23^,^24^.

In this work, we report on the observation of noise-assisted energy transport in a network of capacitively coupled *RLC* oscillators, where *R* stands for resistance, *L* for inductance, and *C* for capacitance. Although in previous studies of ENAQT noise has been modeled as fluctuations in the frequency of each oscillator, so-called diagonal fluctuations^2^, here we introduce noise in the system by means of stochastic fluctuations in one of the network’s capacitive couplings, referred to as off-diagonal dynamical disorder^25^. Using this system, we show that fluctuations in the coupling can indeed influence the system so that the energy transferred to one of the oscillators is increased, demonstrating that off-diagonal dynamical disorder can effectively be used for enhancing the efficiency of energy transport systems.

## Results

**Figure 1:**
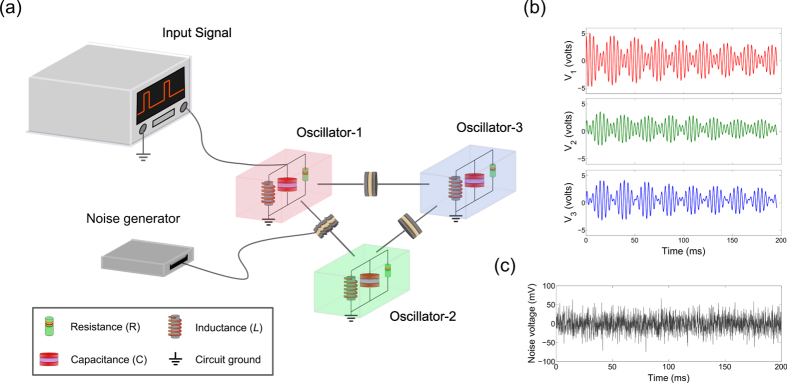
Schematic representation of the experiment and typical dynamic interaction response. (**a**) Schematic of the network of three capacitively coupled *RLC* electrical oscillators. (**b**) Typical averaged voltage signals measured in each oscillator. (**c**) Sample of a typical noise signal, *V*_*s*_, introduced in the capacitive coupling between the first and second oscillator.

We consider a network of three identical *RLC* oscillators (as shown in Fig. 1), whose dynamics are described by


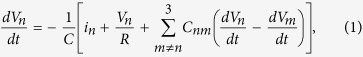



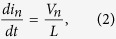


where *V*_*n*_ is the voltage in each oscillator, *i*_*n*_ is the current, and *C*_*nm*_ represents the capacitive couplings.

Noise is introduced in the system by inducing random fluctuations in one of the capacitive couplings, so that


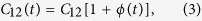


with *C*_12_ being the average capacitance of the coupling and *ϕ*(*t*) a Gaussian random variable with zero average, i.e. 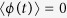, where 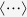 denotes stochastic averaging.

Previous studies of noise-assisted transport have shown that efficiency enhancement can be observed by measuring the energy that is irreversibly dissipated in one particular site of the network, the so-called sink or reaction center^3^,^8^,^14^,^17^. Here, we take the resistance in Oscillator-2 to be the sink and measure the relative energy that is dissipated through it by computing


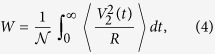


where 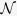 is the total energy dissipated by all the oscillators. Notice that Eq. (4) is equivalent to the efficiency measure that was derived in a previous work on noise-assisted transport in classical oscillator systems^17^.

We have experimentally implemented the system described by Eqs. (1, 2, 3) using functional blocks synthesized with operational amplifiers and passive linear electrical components (see Methods and accompanying Supplementary Information). Our experimental setup was designed so that the frequencies of each oscillator were the same (*v* = 290.57 Hz), as well as the couplings between them (*C*_*nm*_ = 40 *μ*F). Notwithstanding, based on our measurements, we found that the designed system was characterized by the following parameters: *ν* = 283.57 Hz, *C*_12_ = 39 *μ*F, *C*_13_ = 41.7 *μ*F, and *C*_23_ = 39 *μ*F. These variations in the parameters of the system result from the tolerances (around 5%) of all the electronic components used in the implementation of the circuit.

**Figure 2:**
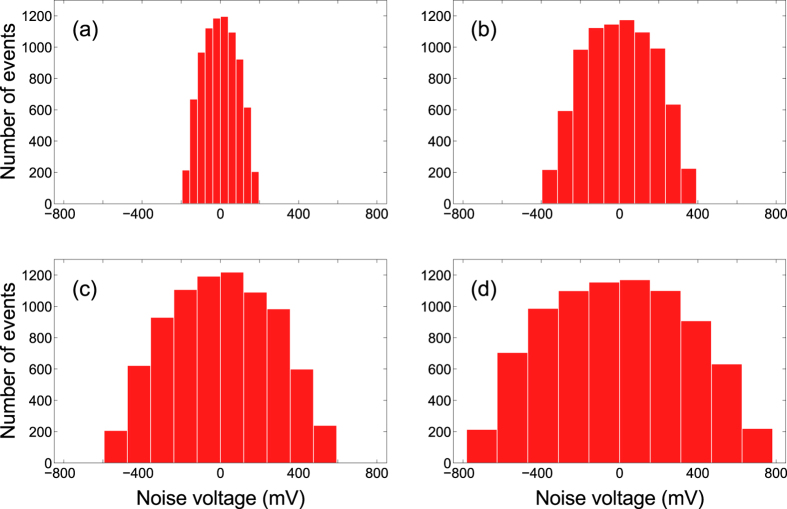
Histograms of noise signals extracted directly from the arbitrary function generator using different values of noise voltage: (**a**) *V*_*s*_ = 200 mV, (**b**) *V*_*s*_ = 400 mV, (**c**) *V*_*s*_ = 600 mV and (**d**) *V*_*s*_ = 800 mV. Samples were obtained from noise signals with frequency of 1 kHz, within a 1 s time-window. Number of events is defined as the number of samples that have the same voltage.

As described in the methods section, noise was introduced in one of the network’s capacitive couplings using a random signal provided by a function generator (Diligent Analog Discovery 410-244P-KIT). The electronic circuit was designed so the voltage of the noise signal, *V*_*s*_, is directly map into Eq. (3), thus making the stochastic variable *ϕ*(*t*) fluctuate within the interval (−*V*_*s*_, +*V*_*s*_), with a 1 kHz frequency. Notice that the frequency of the noise signal is higher than the oscillators’ natural frequency, which guarantees a true dynamical variation of the capacitive coupling. Figure 2 shows some examples of the histograms of noise signals extracted directly from the function generator.

**Figure 3:**
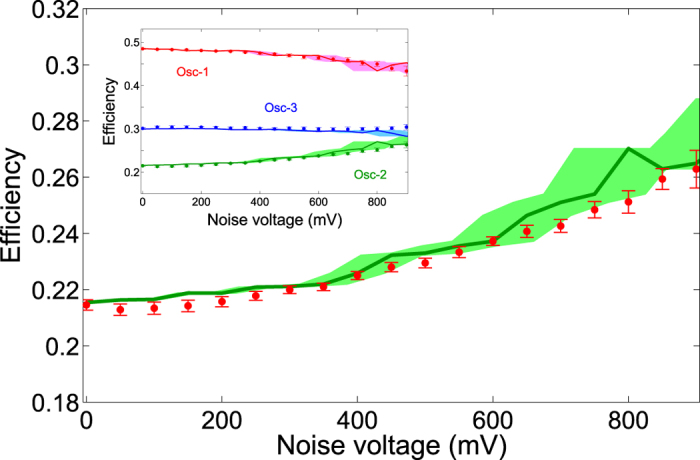
Transport efficiency measured in Oscillator-2 as a function of the noise voltage. Experimental results (dotted line) were obtained by averaging the oscillator’s signal over 500 stochastic realizations. The solid line represents the theoretical calculation of transport enhancement using the noise signal extracted directly from the arbitrary function generator. The shaded region represents transport enhancement when noise introduced in the capacitive coupling deviates from the value provided by the function generator by up to 10%. Transport efficiencies of all oscillators are plotted in the inset. Notice that the effect of noise is to rearrange the energy available in the system in order to increase the efficiency of Oscillator-2. Error bars correspond to one standard deviation.

Using the configuration described above, we measured the energy dissipated through the resistor in Oscillator-2 [Eq. (4)] as a function of the noise voltage introduced in the capacitive coupling. We can see from Fig. 3 that transport efficiency in Oscillator-2 is enhanced as the noise voltage increases, a sign of noise-assisted energy transport^17^. We obtained an enhancement of 22.50 ± 3.59% for a maximum noise voltage of 850 mV. It is important to remark that we cannot go beyond this value using the present configuration because, when introducing noise signals close to 1 volt (or more), the system becomes unstable due to the presence of negative capacitances in the coupling [see Eq. (3)]. However, as obtained in our numerical simulations, a higher efficiency may be reached by incorporating random fluctuations in the remaining couplings.

To verify that the observed enhancement was a consequence of energy rearrangement due to random fluctuations in the coupling, and not because external energy was introduced in the electronic circuit, we measured the transport efficiency of all oscillators. We can see from the inset in Fig. 3 that transport efficiency of Oscillator-2 is enhanced only because the energy dissipated through Oscillator-1 becomes smaller. This clearly shows that the effect of noise is to create new pathways in the system through which energy can efficiently flow towards an specific site, generally referred to as sink or reaction center.

## Discussion

The results presented here show that noise-assisted transport, an ubiquitous concept that may help us understand efficient energy transport in diverse classical and quantum systems, can be observed in simple electronic circuit networks. This opens fascinating routes towards new methods for enhancing the efficiency of different energy transport systems, from small-scale RF and microwave electronic circuits to long-distance high-voltage electrical lines. In this way, a specific feature initially conceived in a quantum scenario (environment-assisted quantum transport) has shown to apply as well in classical systems, widening thus the scope of possible quantum-inspired technological applications.

## Methods

In our experiment, three identical *RLC* electrical oscillators interact by means of three ideal capacitors. The input energy is injected into a single oscillator (Oscillator-1), and we follow the dynamics of the system by registering independently the voltage of each oscillator (Fig. 1). Noise is introduced in the system by means of random fluctuations in one of the capacitive couplings, where the magnitude of the changes in the capacitance is defined by the noise voltage provided by an arbitrary function generator (Diligent Analog Discovery 410-244P). Relative changes in the capacitance range from 0% (no voltage) to 100% (1 V) in a controllable way. To avoid instabilities, the 1-KHz-frequency noise signal was varied from 0 to 850 mV, which corresponds to a range of the fluctuating capacitance going from *C*_12_ to 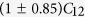.

The voltage of each oscillator contains the information about the stored energy in the system as well as the energy dissipated by each oscillator. A Tektronix MSO4034 oscilloscope (impedance 1MΩ) is used to measure these voltages. The voltage signals were extracted from the oscilloscope using a PC-OSCILLOSCOPE interface, which transfers the information through a USB port. Because we are working with stochastic events, measurements were repeated up to 500 times for each noise voltage, and averaged using a MatLab script. The initial input signal is a single pulse, with a pulse duration of 200 *μ*s, injected in Oscillator-1 using an Arbitrary Waveform Generator from Agilent 33220 A, with a 5 Hz frequency. The high level voltage amplitude is 5 V, while the low-level voltage amplitude is 0 V.

The synthesized transformation of the electronic circuit to an analog computer is described in the Supplementary Information. Basically, the analog computer makes analog simulations of differential equations using electronic components. The input and output voltages of an electronic circuit correspond to mathematical variables. These voltage variables are therefore representations of the physical variables used in the mathematical model.

An important issue that we would like to point out is that in this work both, the initial conditions and noise, are physically introduced via a voltage signal. This is particularly relevant because in the first case this voltage represents the initial current and, in the second case, the statistical distribution of the noise voltage can be defined independently from the circuit, thus it is not necessary to produce fluctuations in the physical properties of the electrical components–namely resistors, capacitors or inductors–which generally represents a major challenge^26^.

Using the building blocks described in the Supplementary Information, it is possible to synthesize current variables and inductor elements with only resistors, capacitors and voltages. In our experiment, the components employed for the implementation of the corresponding building blocks include metal resistors (1% tolerance), polyester capacitors and general-purpose operational amplifiers (MC1458). A DC power source (BK Precision 1761) was used to generate a ±12 V bias voltage for the operational amplifiers. The electronic components of each oscillator were mounted and soldered on a drilled phenolic board (7.5 × 4.5 cm) to avoid poor contacts.

## Additional Information

**How to cite this article**: León-Montiel, R. J. *et al.* Noise-assisted energy transport in electrical oscillator networks with off-diagonal dynamical disorder. *Sci. Rep.* **5**, 17339; doi: 10.1038/srep17339 (2015).

## Supplementary Material

Supplementary Information
