# Supplementary material for: Noise-assisted energy transport in electrical oscillator networks with off-diagonal dynamical disorder
Source: Sci Rep. 2015 Nov 27;5:17339. doi: 10.1038/srep17339 (PMC4661523; doi:10.1038/srep17339)
Supplement: Supplementary Information [file srep17339-s1.pdf]

Supplementary Information:
Noise-assisted energy transport in electrical oscillator networks with off-diagonal dynamical disorder

Roberto de J. León-Montiel, Mario A. Quiroz-Juárez, Rafael Quintero-Torres, Jorge L. Domínguez-Juárez, Héctor M. Moya-Cessa, Juan P. Torres and José L. Aragón

From an experimental point of view, it is challenging to implement a set of interacting *RLC* electrical oscillators where certain important defining parameters should change dynamically (noise), even more if the time needed to visualize the dynamics and interactions is large compared with the time-constant imposed by the parasitic attenuation of a real system. In this work we are interested in noise, its implementation, its effects and how to control it. In the first instance, one could attempt to build noisy *RLC* oscillators using passive components,[1] but this strategy is in general limited by the large damping coefficient in the inductor, which produces a fast energy extinction, thus hindering the observation of the sought-after effects.

In this supplementary information material, we will show how a network of noisy *RLC* oscillators can be electronically implemented by using the working principle of an analog computer.[2,3] Basically, an analog computer makes analog simulations of differential equations using electronic components. The input and output voltage variables of an analog electronic circuit (analog computer) represent physical variables of the mathematical model that is being studied. In this way, in order to simulate any mathematical model in an analog computer, the sequence of mathematical operations involved in the process must be described by means of functional block diagrams. The four functional blocks shown in Fig. 1 are the ones used in our experimental setup to implement the mathematical operations described by Eqs. (1)-(3) of the main manuscript.

Using these functional blocks a complete diagram of the oscillator network can be obtained. Firstly, we build a block diagram for a simple *RLC* oscillator [as shown in Fig. 2(a)]. Notice that this diagram corresponds to the synthesis of a typical damped harmonic oscillator equation, with α and ω representing its damping coefficient and frequency, respectively. Once the block diagram is built, each mathematical operation can be electronically implemented with passive linear electrical components and basic inverting configurations of operational amplifiers (OPAMPs).

Figure 2(b) shows the synthesized analog circuit for a typical RLC electrical oscillator. Notice that the inherent sign inversion is a result of the negative voltage gain of the OPAMP, which must be taken into account in the design. Here, R_j, C_j and U_j stand for resistors and capacitors, and general-purpose operational amplifiers, respectively. The values of $R_{f1}, R_1, R_{i1}, C_{i1}, R_{i2}$ and C_{i2} are defined by the oscillator parameters, that is, the resistance (R), the inductance (L) and the capacitance (C). They must satisfy:

$$\frac{R_{f1}}{R_1} = \frac{1}{R}, \qquad \frac{1}{R_{i1}C_{i1}} = \frac{1}{C}, \qquad \frac{1}{R_{i2}C_{i2}} = \frac{1}{L}, \tag{1}$$

and

$$\frac{R_{f2}}{R_2} = \frac{R_{f2}}{R_3} = \frac{R_{f2}}{R_4} = 1. \tag{2}$$

This last expression [Eq. (2)] implies that the adder (U_4) has a unitary amplification factor.

For the experimental setup, we calculated the resistors and capacitors involved in Fig. 2(b) considering the following parameters: $L = 1$ mH, $C = 300$ μF and $R = 1$ kΩ. The combination of these parameters results in an oscillation frequency of $f = 290.57$ Hz, with a damping coefficient $\alpha = 3.333$ Hz. To design this configuration we thus take $R_{i1} = 3$ kΩ, $C_{i1} = 100$ nF, $R_{i2} = 10$ KΩ, $C_{i2} = 100$ nF, $R_1 = 1$ kΩ and $R_{f1} = 3.3$ kΩ. By selecting $R_{f2} = R_2 = R_3 = R_4 = 10$ kΩ, the condition given by Eq. (2) is satisfied.

To understand how random fluctuations in the coupling between Oscillator-1 and Oscillator-2 are introduced, we first substitute Eq. (3) into Eqs. (1) and (2) of the main manuscript, so we obtain a set of equations given by

$$CV_1' = -i_1 - \frac{V_1}{R} - C_x(V_1' - V_2')(1+\phi) - C_xV_1' + C_xV_3', \tag{3}$$

$$Li_1' = V_1, \tag{4}$$

$$CV_2' = -i_2 - \frac{V_2}{R} + C_x(V_1' - V_2')(1+\phi) - C_xV_2' + C_xV_3', \tag{5}$$

$$Li_2' = V_2, \tag{6}$$

$$CV_3' = -i_3 - \frac{V_3}{R} - 2C_xV_3' + C_xV_1' + C_xV_2', \tag{7}$$

$$Li_3' = V_3, \tag{8}$$

where we have assumed that the coupling capacitors have the same value, that is, $C_x = C_{12} = C_{13} = C_{23}$.

The synthesis of Eqs. (3)-(8) is divided into three parts. In the first part, the *RLC* oscillators are implemented using the electronic circuit in Fig. 2. Each oscillator can be easily implemented using the same electronic circuit that was obtained for the simple *RLC* parallel oscillator. In the second part, the couplings without noise are built using the same methodology, that is, by designing the block diagram and then replacing each block with electronic configurations based on operational amplifiers. Finally, the third part is the design of the noisy coupling. Note that $CV_1^{'}$ and $CV_2^{'}$ have the same noisy coupling term thus it is enough to build only one of them. Figures 3(a)-(b) show the functional block diagram of the noisy coupling, as well as its equivalent analog electronic circuit.

To conclude, it is important to point out that, in our system, noise is physically introduced via a voltage signal. This is particularly relevant because its statistical distribution will be defined independently from the circuit, which implies that fluctuations in the physical properties of electronic components–such as resistors, capacitors or inductors–are not required.

References

[1] León-Montiel, R. de J., Svozilík, J. & Torres, J. P. Generation of a tunable environment for electrical oscillator systems. *Phys. Rev. E* **90**, 012108 (2014).

[2] The Education and Training Department. Carlson, A., Hannauer, G., Carey, T. & Holsberg, J. (Eds) *Handbook of analog computation* (Electronic Associates Inc., New Jersey, 1967) Associates.

[3] Johnson, C. L. *Analog computer techniques* (McGraw-Hill, New York, 1963)

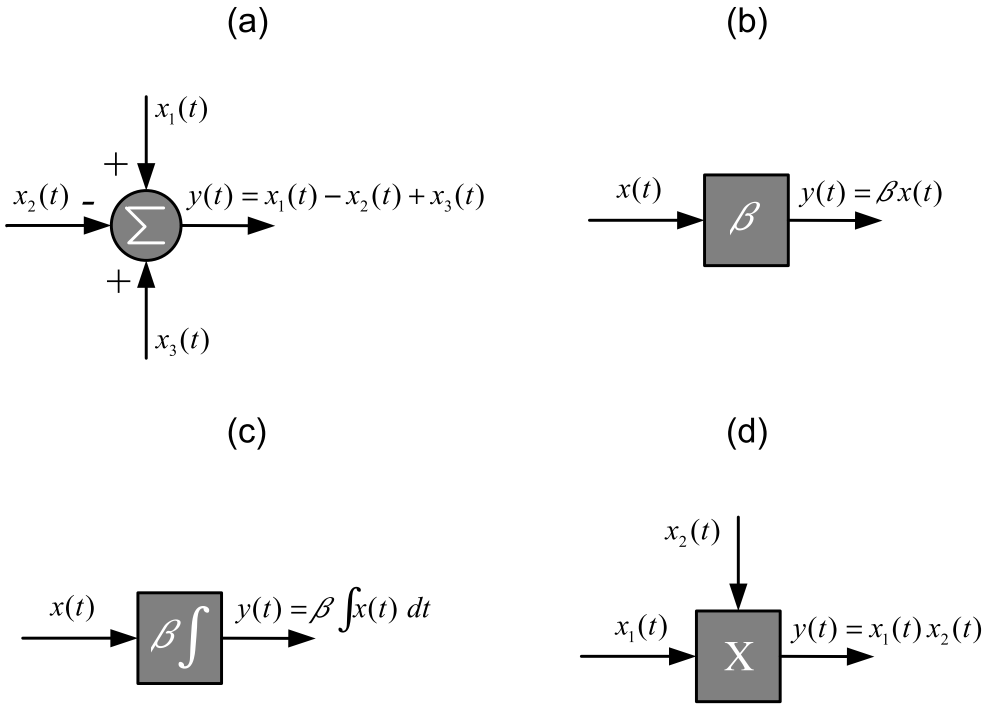

Figure 1: Functional Blocks: (a) adder, (b) multiplication by a constant, (c) integrator and (d) multiplication of signals.

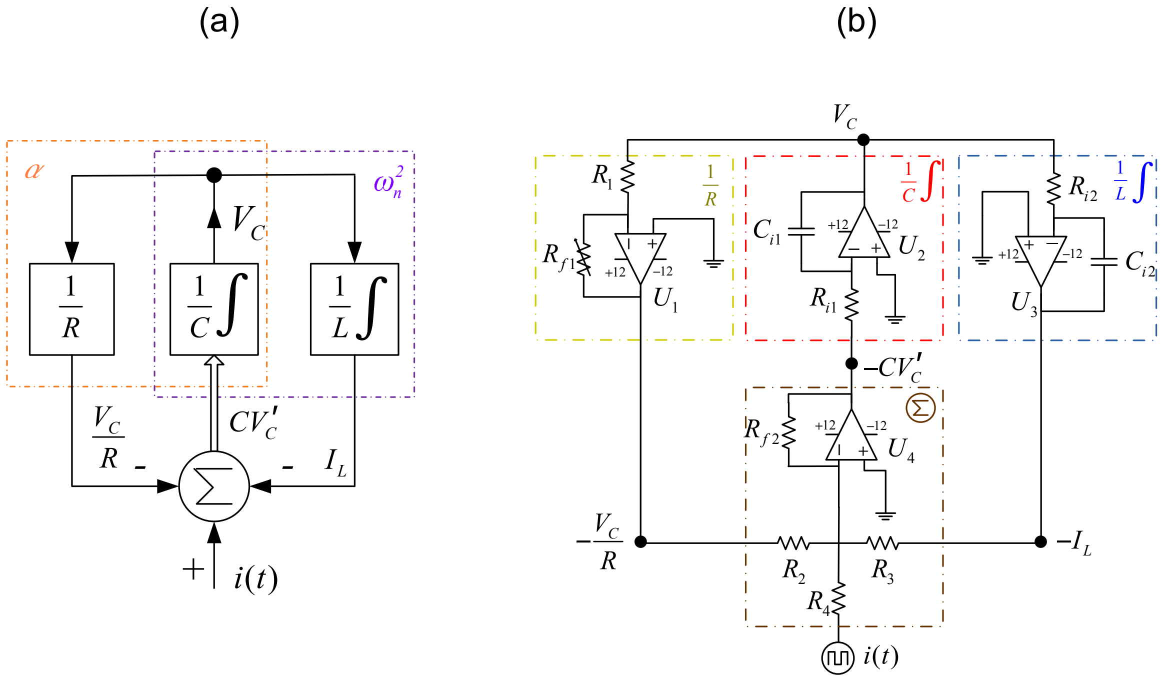

Figure 2: Simple *RLC* parallel oscillator (a) Block diagram and (b) Analog electronic circuit.

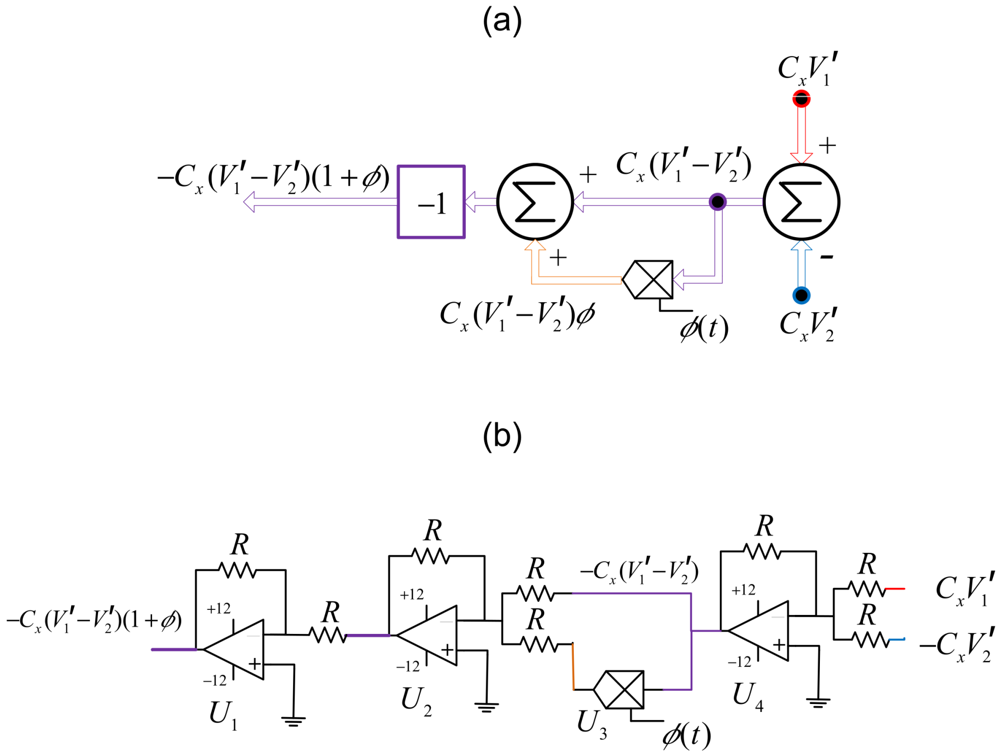

Figure 3: Noisy coupling between Oscillator-1 and Oscillator-2: (a) Block diagram and (b) Analog electronic circuit.
